# Antimicrobial Activity against *Paenibacillus larvae* and Functional Properties of *Lactiplantibacillus plantarum* Strains: Potential Benefits for Honeybee Health

**DOI:** 10.3390/antibiotics9080442

**Published:** 2020-07-24

**Authors:** Massimo Iorizzo, Bruno Testa, Silvia Jane Lombardi, Sonia Ganassi, Mario Ianiro, Francesco Letizia, Mariantonietta Succi, Patrizio Tremonte, Franca Vergalito, Autilia Cozzolino, Elena Sorrentino, Raffaele Coppola, Sonia Petrarca, Massimo Mancini, Antonio De Cristofaro

**Affiliations:** 1 Department of Agriculture, Environmental and Food Sciences, University of Molise, 86100 Campobasso, Italy; iorizzo@unimol.it (M.I.); bruno.testa@unimol.it (B.T.); silvia.lombardi@unimol.it (S.J.L.); m.ianiro@studenti.unimol.it (M.I.); francesco.letizia@unimol.it (F.L.); succi@unimol.it (M.S.); tremonte@unimol.it (P.T.); franca.vergalito@unimol.it (F.V.); autilia.cozzolino@unimol.it (A.C.); sorrentino@unimol.it (E.S.); coppola@unimol.it (R.C.); maxman@unimol.it (M.M.); decrist@unimol.it (A.D.C.); 2 Consorzio Nazionale Produttori Apistici CONAPROA, 86100 Campobasso, Italy; sonia_petrarca@libero.it

**Keywords:** *Lactiplantibacillus plantarum*, probiotics, *Paenibacillus larvae*, honeybee

## Abstract

*Paenibacillus larvae* is the causative agent of American foulbrood (AFB), a severe bacterial disease that affects larvae of honeybees. The present study evaluated, in vitro, antimicrobial activity of sixty-one *Lactiplantibacillus plantarum* strains, against *P. larvae* ATCC 9545. Five strains (P8, P25, P86, P95 and P100) that showed the greatest antagonism against *P. larvae* ATCC 9545 were selected for further physiological and biochemical characterizations. In particular, the hydrophobicity, auto-aggregation, exopolysaccharides production, osmotic tolerance, enzymatic activity and carbohydrate assimilation patterns were evaluated. The five *L. plantarum* selected strains showed suitable physical and biochemical properties for their use as probiotics in the honeybee diet. The selection and availability of new selected bacteria with good functional characteristics and with antagonistic activity against *P. larvae* opens up interesting perspectives for new biocontrol strategies of diseases such as AFB.

## 1. Introduction

*Paenibacillus larvae*, the causative agent of the quarantine disease American foulbrood (AFB), is the most widespread fatal brood disease of honeybee (*Apis mellifera* L.) larvae and pupae [1]. This gram-positive, flagellated, spore-forming bacterium is highly adapted to honeybee larvae [1,2]. The honeybee gut is the site of *P. larvae* infection, as well as of pathogens such as *Ascosphaera apis*, *Nosema ceranae*, and probably many of the honeybee viruses [3]. Following ingestion, through spore-contaminated food, the spores germinate in the larval midgut lumen, where the vegetative bacteria massively proliferate before eventually breaching the midgut epithelium and invading the hemocoel, causing the death of the larvae, which during their decay releases a large number of spores. In the further course of the disease within the colony, more and more larvae become infected and die so that in the end, the lack of brood and, hence, the lack of progeny leads to collapse of the entire colony [1,2]. *P. larvae* often remains dormant in its spore-form and does not induce manifestations of AFB. It has been suggested that *P. larvae* may exist as a pathobiont in the native microbiota of adult worker bees and is then transmitted throughout the hive to fresh brood cells [4]. The extreme contagiousness of AFB and the lethality for larvae and for entire colonies are the reasons why it is a notifiable disease in most countries. Currently, since an effective therapy against AFB is not available, the authorities consider the burning of infected colonies as the only efficient control measure [5]. Over the last few years, a number of different measures such as the use of chemical fungicides, antibiotics, heterocyclic organic compounds (indoles) and bacteriophages have been tried against AFB disease [6,7,8,9,10]. Unfortunately, these approaches could be useful as therapy, but are often ineffective for prophylactic purposes, and hives remain vulnerable to diseases. Moreover, the prophylactic use of antibiotics has inevitably led to the onset of antibiotic resistance in *P. larvae* [11,12]. In addition, the use of chemical compounds should be limited, both because they are dangerous to honeybee health [13] and because any residues present in honey also pose a serious risk to human health [14]. The use of natural compounds for disease control could represent a more suitable alternative [15,16]. Essential oils and other vegetable extracts from plants, herbs and spices exhibit antimicrobial activity against *P. larvae* [17,18] and this activity is mainly due to the presence of phenolic and terpenoid compounds, which have well-known antimicrobial activity [19,20,21]. However, the effects of these substances on honeybee health and on its symbiotic microflora are not entirely known [22]. Currently, there is an increased interest in investigating new, effective and safe control methods. In this context, the use of probiotic bacteria in the prevention and biocontrol of honeybee pathogenic microorganisms offers interesting perspectives [23]. The use of probiotic bacteria, unlike synthetic or natural chemical compounds, does not adversely affect the balance of gut microbiota and honeybee health [24,25]. Moreover, the protection against pathogens and/or parasites is one of the aspects frequently associated with a balanced intestinal flora [26,27,28]. It is well known that the initial phase of pathogen infection can be facilitated by any nutritional or environmental stress causing microbial dysbiosis [29,30,31]. The presence of lactic acid bacteria (LAB) in the honeybee gastrointestinal tract has been consistently reported in literature [32,33,34]. Beneficial bacteria, belonging to LAB, have been shown to promote honeybee health through activating the honeybee’s immune defenses and producing antimicrobial compounds inhibiting pathogenic microorganisms [35,36,37,38,39,40,41,42,43,44]. The antagonistic effects of symbiotic LAB against *P. larvae* can be exploited to develop a new approach to AFB disease control [45,46,47]. Among LAB, *Lactiplantibacillus plantarum* (formerly *Lactobacillus plantarum*) [48] is a versatile bacterium characterized by a high adaptability to many different conditions, being isolated from various ecological niches including dairy, fruits, cereal crops, vegetables, fish and fresh meat [49]. In addition, its presence in honeybee gut has been documented by several researchers [50,51,52], and its role, along with other bacteria and yeasts, in the transformation of fresh pollen into bee bread is well known [53,54]. *L. plantarum* pro-technological properties are exploited in different agri-food sectors [55,56,57,58,59,60,61,62,63]. Moreover, some strains of *L. plantarum* are known for their ability to produce several natural antimicrobial substances, thus inhibiting competitors that share the same niche [64,65,66,67,68,69]. The natural genomic architecture is the basis of its versatility and of its success in industrial applications, not only as starter culture but also as a bio-protective agent [70]. However, while numerous data on the functional and probiotic properties of *L. plantarum* in the diet of fish and mammals, including humans, were obtained [71,72,73,74,75], to our knowledge, its use as a probiotic in the honeybee diet and its antagonistic action against *P. larvae* has been little studied [76,77,78]. In this research we investigated the inhibitory properties of *L. plantarum* strains, isolated, in previous studies [79], from the honeybee (*A. mellifera L.*) gut and bee bread, against *P. larvae* ATCC 9545. In addition, some of their functional characteristics have been evaluated for a possible probiotication of the sugar syrups, to be used in the supplemental feeding of honeybees.

## 2. Results

### 2.1. Antimicrobial Activity

The antagonistic activity, of sixty-one *L. plantarum* strains, against *P. larvae* ATCC 9545 was investigated. The results of a preliminary screening, using the agar spot test, showed that thirty-five strains did not cause any inhibition and twenty-one strains caused an inhibition zone (ZOI) < 4 mm. P8, P25, P86, P95 and P100 *L. plantarum* strains, demonstrating the greatest antagonism against the pathogen (ZOI > 4 mm), were selected for the subsequent investigations (Table S1, Supplementary Materials). In this further analysis, the antimicrobial activity was carried out using agar well diffusion assay and the inhibitive capacity was assessed using cultural broth (BC) and cell free supernatants (CFS) of the five selected strains. The results of the antimicrobial activity are reported in Table 1.

The BC, of five *L. plantarum,* inhibited *P. larvae* more than CFS. In particular, BC caused a ZOI between 4.2 and 7.2 mm wide, whereas the CFS caused a ZOI between 3.4 and 5.8 mm wide. The highest inhibitive activity was produced by *L. plantarum* P8 strain. The MRS broth at pH 3.5, used as control, did not produce any inhibition.

### 2.2. Exopolysaccharides Production

The exopolysaccharides (EPS) amounts, produced by the five *L. plantarum* selected strains, in MRS broth after 48 h at 37 °C in aerobiosis were generally significantly different among them (Table 1). In particular, P95 strain was the lowest EPS producer (76 mg/L); the other strains produced quantities greater than 130 mg/L with a maximum of 174 mg/L produced by the *L. plantarum* P100 strain.

### 2.3. Cell Surface Properties: Hydrophobicity and Auto-Aggregation

**Figure 1:**
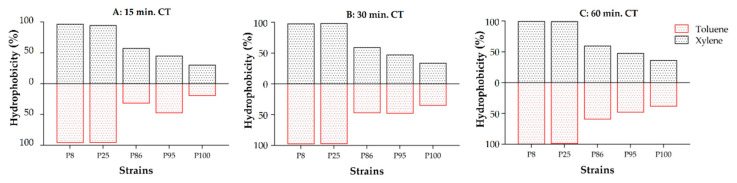
Adhesion of the *L. plantarum* five selected strains to toluene and xylene (expressed as hydrophobicity %) measured using bacterial ability to adhere to hydrocarbons (BATH) test after different contact times (CTs). (**A**) 15 min; (**B**): 30 min; (**C**) 60 min.

The hydrophobicity was assessed using the ability of the bacteria to adhere to toluene and xylene hydrocarbons. The results of the hydrophobicity (%) of the five *L. plantarum* selected strains are reported graphically in Figure 1 and numerically in Table S2 (Supplementary Materials). For each strain, the bacteria adhesion to the two hydrocarbons was similar and increased gradually during the test period (60 min). P8 and P25 strains, already after 15 min, showed a high adherence to toluene and xylene with a hydrophobicity percentage greater than 90%, and after 60 min, the percentage was around 99%. P86 and P95 strain adherence rates, after 60 min, were less than 60% both to xylene and toluene. P100 strain showed the lowest hydrophobicity with adherence values less than 40%, to both hydrocarbons, after 60 min.

**Figure 2:**
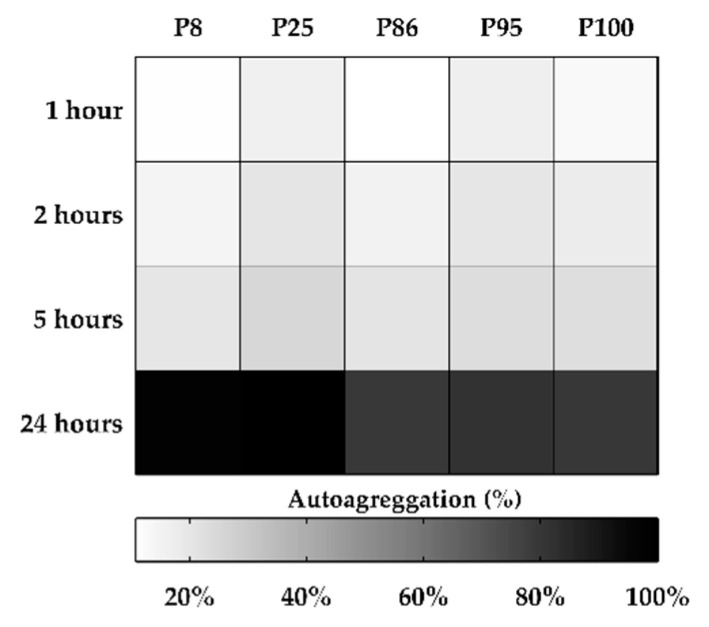
Auto-aggregation (AA%) of the *L. plantarum* strains expressed as optical density (OD) value at 580 nm.

The auto-aggregation (AA%) was assessed by measuring the optical density decrease of the five *Lactiplantibacilli* cultures suspended in phosphate saline buffer (PBS). The analyses showed that the ability to aggregate and sediment increased progressively over time, until reaching, after 24 h, value ranges between 78.78% (P86 strain) and 99.60% (P25 strain) with significant differences among all strains. The AA results are reported graphically in Figure 2 and numerically in Table S3 (Supplementary Materials).

### 2.4. Biochemical Characterization

The results of the enzymatic profile, obtained using an API ZYM kit, are shown in Table 2. P25 and P95 strains did not show esterase-lipase, leucine- and valine-arylamidase and α-fucosidase activity. In addition, P8 strain did not exhibit α-galactosidase and esterase activity, which were detected in the other strains. All the strains exhibited alkaline phosphatase, β-galactosidase, alpha- and β-glucosidase and N-acetyl-β-glucosaminidase activity.

The carbohydrate assimilation patterns, detected using API 50 CHL medium, are shown in Table 3. All the *L. plantarum* five strains showed very similar profiles. Only P8 strain did not ferment the methyl-a-D-mannopyranoside. P25 and P86 strains were able to ferment l-sorbose, and P100 was able to ferment the d-xylose, unlike the other strains.

### 2.5. Bacterial Survival in Sugar Syrup

The bacterial osmotic tolerance was assessed based on survival ability in two different sugar syrups and the results are showed in Table 4. In test A (40% glucose + 20% fructose, pH 4.2), after 24h of storage, the P25 strain exhibited a reduction in cell viable density of about 2 log units, the other strains maintained a high cell density around 7.0 log CFU/mL; after 48 h of storage, the five strains maintained a cell density ranging between 3.22 (P25 strain) and 5.84 (P95 strain) log CFU/mL. In test B, using sugar syrup with 50% of sucrose at pH 4.2, after 24 h of storage, the viable cell density of the P8, P86, P95 and P100 strains remained similar to the initial one and decreased, by about 1 log unit, after 48 h. For the P25 strain, 1 log reduction, after 24 h, and 2 log reduction, after 48 h, of initial viability were detected.

## 3. Discussion

The role that probiotic bacteria can play as antagonists of honeybee pathogens, using the honeybee digestive tract as the site of infection, is very important [23,33,42,43]. The LAB antimicrobial action is often due to different factors: nutritional competition and compounds production as organic acids, fatty acids, proteinaceous compounds, phenolic acids and hydrogen peroxide [80,81]. In the inhibition test, carried out using the agar well diffusion method, the BC of the *L. plantarum* five strains showed a greater antimicrobial activity against *P. larvae* ATCC 9545, compared to that showed by CFS. Our results suggest that *Lactiplantibacilli* antagonistic action is due to different compounds present in BC that could increase overall antimicrobial activity. The absence of inhibition in the control test, carried out with MRS acidified, excludes that the inhibitive action of bacterial cultures is due to a low pH. In addition, the inability of many *L. plantarum* strains to inhibit *P. larvae* ATCC 9545 (Table S1) suggests that the antimicrobial activity is not due to nutritional competition. In this regard, previous research demonstrated that some LABs produce extracellular substances, secreted or tied to the cell wall, which can perform an inhibitory action against competing microorganisms [43,82,83]. Some extracellular polymeric substances (polysaccharides, proteins, nucleic acids and lipids) are responsible for the cohesion of microorganisms and involved in biofilm formation [84]. The EPS production and the biofilm formation by LAB could be an effective strategy against biofilms and colonization of pathogenic bacteria, since they compete with them for nutrients and space with different mechanisms of action [85,86,87,88,89,90,91,92]. Fünfhaus et al. [93] showed that *P. larvae* were able to form biofilms at the beginning of the saprophytic phase, and this could promote optimal colonization of the honeybee larvae cadaver and the access to all nutrients. Several studies reported that some *L. plantarum* strains produce exopolysaccharides that, as well as contributing to biofilm formation, can exert an antimicrobial action [89,94,95,96]. Based on these considerations, we evaluated the ability of selected *L. plantarum* strains to produce extracellular polysaccharides (EPS). Our results showed that in MRS broth at 37 °C, and aerobiosis conditions, all the *L. plantarum* tested produced EPS and, in accordance with other researchers, this ability can be highly variable among *L. plantarum* strains [97]. Furthermore, the strains producing greater amounts of EPS also caused the strongest inhibitory action against *P. larvae* (Table 1). This suggests that there may be a correlation between these two properties. Further studies are needed to investigate EPS composition and to assess its capacity to inhibit spore germination, biofilm formation and vegetative growth of *P. larvae*. In addition to EPS production, also surface hydrophobicity and auto-aggregation are phenotypic traits that favor the biofilm formation and stability of microbial strains in the gastrointestinal tract [98,99]. The adhesion to intestinal epithelial cells is an important prerequisite for colonization of probiotic bacteria, preventing their immediate elimination by peristalsis and providing a competitive advantage in this ecosystem. In our work, we tested the selected strains potentiality to adhere to the intestinal tract, using bacterial adhesion to hydrocarbons (BATH), a method that determines the hydrophobicity or hydrophilic nature of the cell surface [100,101]. The hydrophobicity was assessed by carefully mixing a bacterial culture and hydrocarbon suspension (xylene and toluene) and then the decrease in optical density of the culture phase was evaluated. Based on the adherence % to hydrocarbons, the LAB could be classified into three groups: those with low (0 to 35%), moderate (36 to 70%) and high hydrophobicity (71 to 100%) [102,103]. Under these ranges, in the BATH test, P86, P95 and P100 *L. plantarum* strains showed a moderate hydrophobicity, on the contrary, P8 and P25 showed a high one. The variable values indicate that hydrophobicity appears to be strain-dependent and not species-dependent. In the future, it would be necessary to perform the assay with cell lines to confirm the ability of the selected strains to adhere to epithelial cells. In the environment, microorganisms live as planktonic cells and prefer growing as aggregates. This self-binding is termed auto-aggregation or auto-agglutination. The ability to auto-aggregate (form floccules) of probiotic bacteria is a correlate with adhesion, is a prerequisite for colonization and protection of the gastrointestinal tract and appears to be the first step in the formation of biofilms [104,105,106]. In general, our results highlighted that the five *L. plantarum* strains tested showed a high auto-aggregation ability and that the percentage of aggregated cells increased over time, in accordance with previous results obtained by other researchers, who have conducted similar studies on strains belonging to the same species [107]. Except for the P8 and P25 strains, which showed a similar aggregation capacity, significant differences (*p* < 0.05) were observed among the strains. This confirms that also the AA is a strain-dependent, not species-dependent, phenotypic character [99,108]. In recent years, beekeeping has become a fundamental need to intervene with an additional carbohydrate supplement for bees to integrate insufficient stocks, for spring and autumn stimulation of colonies or to completely replace stocks. The most widely used syrups contain sucrose, glucose and fructose [109,110]. The viability of probiotic organisms is a very important aspect; before resisting the gastrointestinal tract, they must be able to survive during manufacturing and storage of probiotic products in order to express health benefits for the host. In our experimental studies we evaluated the capacity of five *L. plantarum* selected strains to tolerate a high concentration of sucrose (50%) and of glucose and fructose mixture (40 + 20%) at pH 4.2. Honeybees are attracted by high concentrations of sugar syrup, and this behavior becomes important to find a compromise between maximum attractiveness for honeybees and the survival of LAB. The results showed a good osmotic tolerance of all strains in all combinations. This property would ensure a high vitality if bacteria were added in sugar syrups, used as additional food in hives. Metabolic activities of the microbiota are key for symbiotic interactions in the honeybee gut and they have an impact on the health and disease of the host in different ways [111]. Gut microbiota participate in various processes, including defense systems and protection from pathogens, detoxification from harmful molecules, supply of essential nutrients and food digestion [112,113,114,115]. A balanced gut microbiota is necessarily associated with bee health since it provides countless enzymatic activities to break down the complex sugars of the honeybee’s diet [29,113,116]. The enzyme profile, which we studied, showed that the five *L. plantarum* selected strains possess alpha- and beta-glycosidase activities. The beta-glycosidase is capable of hydrolyzing the glycosylated aromatic precursors, releasing odorous compounds including monoterpenes and increasing the bioavailability of antioxidative plant metabolites in honey, beebread and royal jelly [59,61,117,118,119,120,121,122,123,124,125]. In addition, beta-glycosidase is important, because in combination with other enzymes, including cellulase and hemicellulase, it contributes to the hydrolysis of cellulose [126]. Alpha-glycosidase enzyme converts maltose to glucose and is also directly involved, together with alpha-amylase, in the degradation of starch granules [127]. The impact of carbohydrates on bee survival has been studied, and it is well established that bees live longest on syrup containing sucrose, glucose or fructose [128]. Honeybees collect carbohydrate-rich food to support their colonies, and yet, certain carbohydrates present in their diet have been described as toxic because these insects lack the appropriate enzymes for their digestion [129]. These carbohydrates include the monosaccharides mannose, galactose, xylose, arabinose and rhamnose and the oligosaccharides lactose, melibiose, raffinose and melezitose [130,131,132,133,134]. They are contained in natural nectar or derived from pectin hydrolysis or synthesized as melezitose. This sugar, composed of glucose and turanose and produced by aphids, is primary trisaccharide in honeydew, where it can constitute up to 70% of the sugar fraction [135,136]. The results of carbohydrate assimilation tests showed that the P8, P25, P86, P95 and P100 *L. plantarum* strains are able to metabolize arabinose, galactose, lactose, mannose, melibiose, melezitose and raffinose, considered potentially toxic to honeybees. Given their ability to simultaneously participate in the breakdown of complex polysaccharides and metabolize toxic sugars, the role of these *L. plantarum* strains in improving dietary tolerance as well as maintaining the health of their hosts might be notable [131,137,138]. The selection and availability of new selected bacteria with good functional characters and with antagonistic activity against *P. larvae* always opens up interesting perspectives for new biocontrol strategies of diseases such as the AFB. Some researchers have highlighted the effectiveness of LAB in controlling this disease [45,46,47,76,77,78,139,140], and other researches have shown that effectiveness is not always certain in the hive [141,142] or that supplementation of honeybee diet, with improper probiotics, can be harmful to honeybees [143,144]. The functional properties, shown in vitro using *L. plantarum* strains, do not result axiomatically in health benefits for honeybee colonies. It is therefore necessary to assess in the future, in vivo/in situ, the role that these bacteria can have in maintaining the well-being of bees, and in particular, it is necessary to assess the contribution they can make in a prophylactic strategy against AFB disease.

## 4. Materials and Methods

### 4.1. Microbial Cultures

For this study sixty-one *L. plantarum* strains, isolated from bee bread and honeybee gut of *Apis mellifera* L., were used (Table S1, Supplementary Materials) [79]. These bacteria belong to the Di.A.A.A (Department of Agricultural, Environmental and Food Sciences) collection of the University of Molise. In antimicrobial tests, *P. larvae* ATCC 9545 strain was used as indicator.

### 4.2. Screening of Antibacterial Activity

Sixty-one *L. plantarum* strain antimicrobial activity against *P. larvae* ATCC 9545 was investigated, using agar spot tests. The experiments were conducted by spotting 10 μL of 16 h LAB cultures (10^8^ UFC/mL) onto the surface of MRS (Oxoid Ltd., Hampshire, UK) agar plates, which were then incubated anaerobically at 37 °C for 24 h, to allow colonies to develop. *P. larvae* was cultured in 10 mL of brain heart infusion (BHI-Oxoid Ltd., Hampshire, UK) at 37 °C for 16 h. Subsequently, 100 μL of overnight culture (10^7^ UFC/mL) were inoculated into 7 mL of BHI soft agar (0.7% agar) maintained at 45 °C and poured over the MRS plates on which the selected *L. plantarum* were grown. The plates were incubated aerobically at 37 °C. The tests were conducted in triplicate, and after 48 h, the inhibition was evaluated by measuring the width (mm) of the clear zone (ZOI) around the colonies of the *L. plantarum* strains.

### 4.3. Determination of Antibacterial Activity

The *L. plantarum* strains producing a ZOI greater than 4 mm, in the agar spot test, were selected, and their antimicrobial activity against *P. larvae* ATCC 9545 was tested using agar well diffusion assay. The *L. plantarum* strains were grown in MRS broth and, after 16 h at 37 °C, the cultural broth (BC) of every single strain was centrifugated (8000 rpm for 20 min at 4 °C) and the supernatant (CFS) was sterilized by filtration (cellulose acetate membrane, pore size 0.22 μm, Sigma-Aldrich; St. Louis, Missouri, USA). The antimicrobial activity of the selected strains was evaluated according to Tremonte et al. protocol [65]. Briefly, 20 mL of BHI soft agar (0.7% agar) inoculated with an overnight culture of *P. larvae* (final concentration of about 10^7^ CFU/mL) were poured into Petri plates. Wells of 5.0 mm in diameter were bored into a single plate and 50 μL of BC and of CFS, of each producer strain, were placed into different wells. As control, 50 μL of MRS, adjusted to pH 3.5 with hydrochloric acid 1N (Sigma-Aldrich), were used. After incubation at 37 °C for 48 h, the plates were observed and antibacterial activity was reported as width (mm) of clear zone of inhibition (ZOI) around the inoculated wells [19,81]. The tests were conducted in triplicate.

### 4.4. Biochemical Characterization

*L. plantarum* strains have been assessed for their carbohydrate fermentation pattern, using an API 50CHL system kit, and for enzymatic patterns, using an API ZYM system kit, according to the manufacturer’s instructions (bioMérieux SA, Marcy l’Etoile, France).

### 4.5. Auto-Aggregation

The auto-aggregation assay was performed according to Cozzolino et al. [145]. Briefly, the *Lactobacilli* cultures were collected using centrifugation (8000 rpm for 10 min at 4 °C) during the logarithmic growth phase. Subsequently, the cells were washed three times with phosphate saline buffer (PBS, Sigma-Aldrich). Further, they were washed twice and re-suspended in PBS to an optical density (OD) of approx. 0.5 (A_580_), in order to standardize the bacterial concentration at 10^8^ CFU/mL. The tests were conducted in triplicate and the cell auto-aggregation was measured at 1, 2, 5 and 24 h of incubation at 37 °C, after which the OD at 580 nm of the upper suspension was measured using a spectrophotometer (Multilabel Counter-PerkinElmer 1420, San Jose, USA). The percentage of auto-aggregation was calculated using the following formula: Auto-aggregation % (A) = (1 − OD_t_/OD_0_) × 100, where OD_0_ is the absorbance at time 0, and OD_t_ is the absorbance detected after 1, 2, 5, and 24 h.

### 4.6. Cell Surface Hydrophobicity

The determination of cell surface hydrophobicity, based on the bacterial ability to adhere to hydrocarbons (BATH), was evaluated on *L. plantarum* strains, using xylene and toluene (Sigma-Aldrich) [145]. Hydrophobicity was calculated as the percentage decrease in OD of the initial bacterial suspension and was expressed using the following formula: % Hydrophobicity = (OD_0_ − OD_t_/OD_0_) × 100, where OD_t_ represents the absorbance value after extraction with hydrocarbons (15, 30 and 60 min), and OD_0_ represents the absorbance value before extraction with hydrocarbons. The tests were conducted in triplicate.

### 4.7. Exopolysaccharides Production (EPS)

For exopolysaccharide production, 100 mL of MRS was inoculated with a 1% (*v*/*v*) of overnight pre-culture. After incubation, at 37 °C for 48 h, cells were separated using centrifugation at 7000 rpm for 30 min at 4 °C. Trichloroacetic acid was added to the supernatant, to a final concentration of 7% (*w*/*v*), which was then incubated at 4 °C for 12 h. The precipitated proteins were removed using centrifugation at 12,000 rpm for 30 min at 4 °C. The supernatant was then mixed with three volumes of pre-chilled ethanol (95%), vigorously stirred and kept at 4 °C overnight. EPS sediments were collected using centrifugation at 17,000 rpm for 30 min at 4 °C. As control, 100 mL of MRS broth without bacterial inoculum were used. The final polysaccharide fractions were lyophilized and their amount was determined by measuring the weight. The net quantity was obtained by subtracting the amount of EPS obtained from non-inoculated MRS broth. The tests were conducted in triplicate. All the chemical compounds used were supplied by Sigma-Aldrich.

### 4.8. Bacterial Survival in Sugar Syrup

To assess osmotic tolerance, a test was performed according to Iorizzo et al. [44], with some modifications. *L. plantarum* strains were grown in MRS broth at 37 °C and after 24 h the cells were harvested using centrifugation at 8000 rpm for 10 min at 4 °C. The fresh pellets were washed twice with PBS buffer and were inoculated in sugar syrup in order to obtain an initial concentration of 10^7^ CFU/mL. The experimental conditions were the following; test A: sugar syrup constituted by 40% glucose + 20% fructose (*w*/*v*) in distilled water at pH 4.2; test B: sugar syrup constituted by 50% (*w*/*v*) sucrose in distilled water at pH 4.2. The sugar syrup was acidified using HCl 1N and sterilized using filtration (cellulose acetate membrane, pore size 0.22 μm, Sigma-Aldrich). The experiments, conducted in triplicate, were performed at 20 °C and the bacterial viability was determined after 0, 24 and 48 h by plating in MRS agar (37 °C for 72 h in anaerobic condition).

### 4.9. Statistical Analysis

All data, obtained by three independent experiments, were expressed as mean ± standard deviation (SD). Statistical analysis was performed through the analysis of variance (ANOVA) followed by the Tuckey’s multiple comparison. Statistical significance was attributed to *p*-values < 0.05. The software SPSS (IBM SPSS Statistics 21) was used for the analysis.

## 5. Conclusions

The results of our research have shown that P8, P25, P86, P95 and P100 *L. plantarum* strains are able to inhibit *P. larvae* ATCC 9545 and they own some physiological and functional properties that make these strains candidate as a probiotic for the honeybee. However, we may consider the preliminary and preparatory results for future studies that can consolidate the acquired knowledge and assess the benefits these bacteria may have on honeybee health in the hive. In addition, it will be important to investigate the factors that determine antimicrobial action and to evaluate antagonist activity of *L. plantarum* strains in vivo/in situ.

## Figures and Tables

**Table 1:** Antimicrobial activity (inhibition zone mm) of *L. plantarum* strains using cultural broths (BC) and cell free supernatants (CFS). Exopolysaccharides (EPS) amounts (µg/mL) in MRS broth after 48 h at 37 °C in aerobiosis. Results are shown as mean ± standard deviation (*n* = 3). Different uppercase letters (A–D), in each column, and different lowercase letters (a,b), in each row, indicate significant differences (*p* < 0.05).

*L. plantarum* Strains	Inhibition Zone (mm)		EPS Production (µg/mL)
	BC	CFS	EPS
**P8**	7.2 ± 0.2 ^Db^	5.8 ± 0.3 ^Ca^	174.0 ± 6.0 ^C^
**P25**	6.0 ± 0.2 ^Cb^	3.7 ± 0.1 ^Aa^	140.0 ± 6.0 ^B^
**P86**	6.1 ± 0.4 ^Cb^	4.1 ± 0.3 ^Aa^	167.0 ± 4.0 ^C^
**P95**	4.2 ± 0.3 ^Ab^	3.4 ± 0.1 ^Aa^	76.0 ± 3.0 ^A^
**P100**	5.1 ± 0.2 ^Bb^	4.6 ± 0.3 ^Ba^	135.0 ± 4.0 ^B^

**Table 2:** Enzymatic profile of the five *L. plantarum* strains performed using API-ZYM system (BioMèrieux). + positive; − negative.

Enzyme Assayed	*L. plantarum* Strains				
	P8	P25	P86	P95	P100
Alkaline phosphatase	+	+	+	+	+
Esterase (C4)	−	+	+	+	+
Esterase lipase (C8)	+	−	+	−	+
Lipase (C14)	−	−	−	−	−
Leucine arylamidase	+	−	+	−	+
Valine arylamidase	+	−	+	−	+
Cystine arylamidase	−	−	−	−	−
Trypsin	−	−	−	−	−
α-chymotryspin	−	−	−	−	−
Acid phosphatase	−	−	−	−	−
Naphthol-AS-BI-phosphohydrolase	+	−	−	−	−
α-galactosidase	−	+	+	+	+
β-galactosidase	+	+	+	+	+
β-glucuronidase	−	−	−	−	−
α-glucosidase	+	+	+	+	+
β-glucosidase	+	+	+	+	+
N-acetil-β-glucosaminidase	+	+	+	+	+
α-mannosidase	−	−	−	−	−
α-fucosidase	−	−	+	−	+

**Table 3:** Carbohydrate assimilation patterns of the five *L. plantarum* strains, performed using API 50 CHL system kit. + positive; − negative.

**Carbohydrates**	***L. Plantarum* Strains**				
	**P8**	**P25**	**P86**	**P95**	**P100**
Glycerol	−	−	−	−	−
Erythritol	−	−	−	−	−
D-arabinose	−	−	−	−	−
L-arabinose	+	+	+	+	+
D-Ribose	+	+	+	+	+
D-Xylose	−	−	−	−	+
L-Xylose	−	−	−	−	−
D-adonitol	−	−	−	−	−
Methyl-b-D-Xylopyranoside	−	−	−	−	−
D-Galactose	+	+	+	+	+
D-Glucose	+	+	+	+	+
D-Fructose	+	+	+	+	+
D-Mannose	+	+	+	+	+
L-Sorbose	−	+	+	−	−
L-Rhamnose	−	−	−	−	−
Dulcitol	−	−	−	−	−
Inositol	−	−	−	−	−
D-Mannitol	+	+	+	+	+
D-Sorbitol	+	+	+	+	+
Methyl-a-D-Mannopyranoside	−	+	+	+	+
Methyl-a-D-Glucopyranoside	−	−	−	−	−
N-Acetyl-Glucopyranoside	+	+	+	+	+
Amygdaline	+	+	+	+	+
Arbutine	+	+	+	+	+
Esculine citrate de fer	+	+	+	+	+
Salicine	+	+	+	+	+
D-Cellobiose	+	+	+	+	+
D-Maltose	+	+	+	+	+
D-Lactose	+	+	+	+	+
D-Melibiose	+	+	+	+	+
D-Saccharose	+	+	+	+	+
D-Trehalose	+	+	+	+	+
Inuline	−	−	−	−	−
**Carbohydrates**	***L. Plantarum* Strains**				
	**P8**	**P25**	**P86**	**P95**	**P100**
D-Melezitose	+	+	+	+	+
D-Raffinose	+	+	+	+	+
Amidon	−	−	−	−	−
Glycogene	−	−	−	−	−
Xylitol	−	−	−	−	−
Gentiobiose	+	+	+	+	+
D-Turanose	+	+	+	+	+
D-Lyxose	−	−	−	−	−
D-Tagatose	−	−	−	−	−
D-Fucose	−	−	−	−	−
L-Fucose	−	−	−	−	−
D-Arabitol	−	−	−	−	−
L-Arbitol	−	−	−	−	−
Potassium Gluconate	−	−	−	−	−
potassium 2-Cetogluconate	−	−	−	−	−
potassium 5-Cetogluconate	−	−	−	−	−

**Table 4:** Survival of the *L. plantarum* strains in sugar syrups stored for 24‒48 h at 20 °C. Test A: 40% glucose + 20% fructose, pH 4.2; test B: 50% sucrose, pH 4.2. Results are shown as mean ± standard deviation (*n* = 3). For every sugar syrup, different uppercase letters (A–C), in each column, and different lowercase letters (a–d), in each row, indicate significant differences (*p* < 0.05).

Storage Time (h)	Sugar Syrup Composition	Survival (log CFU/mL) of *L. Plantarum* Strains				
		P8	P25	P86	P95	P100
T_0_	A 40% glucose 20% fructose	7.30 ± 0.06 ^Ba^	7.29 ± 0.03 ^Aa^	7.32 ± 0.04 ^Ca^	7.34 ± 0.03 ^Ca^	7.30 ± 0.04 ^Ca^
T_24_		7.23 ± 0.02 ^Bb^	5.01 ± 0.04 ^Ab^	7.11 ± 0.02 ^Bb^	7.19 ± 0.04 ^Bb^	7.20 ± 0.05 ^Bb^
T_48_		4.28 ± 0.04 ^Ab^	3.22 ± 0.02 ^Aa^	5.73 ± 0.05 ^Ad^	5.84 ± 0.03 ^Ae^	5.14 ± 0.02 ^Ac^
T_0_	B 50% sucrose	7.23 ± 0.06 ^Ba^	7.15 ± 0.04 ^Ca^	7.22 ± 0.04 ^Ba^	7.20 ± 0.02 ^Ba^	7.29 ± 0.02 ^Ca^
T_24_		7.21 ± 0.05 ^Bc^	6.06 ± 0.05 ^Ba^	7.17 ± 0.03 ^Bc^	7.16 ± 0.02 ^Bc^	7.06 ± 0.06 ^Bb^
T_48_		6.14 ± 0.02 ^Ab^	5.15 ± 0.02 ^Aa^	6.50 ± 0.05 ^Ad^	6.35 ± 0.04 ^Ac^	6.54 ± 0.04 ^Ad^

## Supplementary Materials

The following are available online at https://www.mdpi.com/2079-6382/9/8/442/s1. Table S1: *L. plantarum* strains collection; Table S2: Hydrophobicity (%); Table S3: Auto-aggregation (%).
