# Supplementary material for: Antimicrobial Activity against Paenibacillus larvae and Functional Properties of Lactiplantibacillus plantarum Strains: Potential Benefits for Honeybee Health
Source: Antibiotics (Basel). 2020 Jul 24;9(8):442. doi: 10.3390/antibiotics9080442 (PMC7460353; doi:10.3390/antibiotics9080442)
Supplement: Supplementary file 1 [file antibiotics-09-00442-s001.zip › supp/Supplementary material/Caption-Supplementary material.docx]

**Table S1.** Isolation source and antimicrobial activity of sixty-one *L. plantarum* strains against *P. larvae* ATCC 9545, using agar spot tests.

**Table S2**. Adhesion (expressed as Hydrophobicity %) of the *L. plantarum* strains to hydrocarbons (Xylene and Toluene) measured using the BATH test after 15, 30 and 60 min of contact time (CT). Results are shown as mean ± standard deviation (n = 3). For every hydrocarbon, different uppercase letters (A–C), in each column, and different lowercase letters (a–d), in each row, indicate significant differences (p < 0.05).

**Table S3.** Auto-aggregation (%), of the *L. plantarum* strains, after 1, 2, 5, and 24 h of incubation at 37 °C. Results are shown as mean ± standard deviation (n = 3). Different uppercase letters (A–D), in each column, and different lowercase letters (a–c), in each row, indicate significant differences (p < 0.05).
