# Supplementary material for: Antimicrobial Activity against Paenibacillus larvae and Functional Properties of Lactiplantibacillus plantarum Strains: Potential Benefits for Honeybee Health
Source: Antibiotics (Basel). 2020 Jul 24;9(8):442. doi: 10.3390/antibiotics9080442 (PMC7460353; doi:10.3390/antibiotics9080442)
Supplement: Supplementary file 1 [file antibiotics-09-00442-s001.zip › supp/Supplementary material/Table S1.docx]

| **L. plantarum strains collection** | **Isolation Source** | **ZOI (mm) agar spot test** | **Selected strains** |
| --- | --- | --- | --- |
| P3 | beebread | < 4 mm |  |
| P4 | beebread | no inhibition |  |
| P5 | beebread | < 4 mm |  |
| P7 | beebread | < 4 mm |  |
| P8 | beebread | > 4 mm | P8 |
| P9 | beebread | < 4 mm |  |
| P21 | beebread | < 4 mm |  |
| P36 | beebread | < 4 mm |  |
| P37 | beebread | no inhibition |  |
| P39 | beebread | no inhibition |  |
| P57 | beebread | < 4 mm |  |
| P61 | beebread | no inhibition |  |
| P81 | beebread | no inhibition |  |
| P82 | beebread | < 4 mm |  |
| P86 | beebread | > 4 mm | P86 |
| P87 | beebread | < 4 mm |  |
| P88 | beebread | < 4 mm |  |
| P92 | beebread | < 4 mm |  |
| P94 | beebread | < 4 mm |  |
| P95 | beebread | < 4 mm |  |
| P97 | beebread | < 4 mm |  |
| P101 | beebread | no inhibition |  |
| P103 | beebread | no inhibition |  |
| P106 | beebread | no inhibition |  |
| P108 | beebread | no inhibition |  |
| P1 | honey stomach | no inhibition |  |
| P3 | honey stomach | no inhibition |  |
| P10 | honey stomach | < 4 mm |  |
| P15 | honey stomach | no inhibition |  |
| P21 | honey stomach | no inhibition |  |
| P24 | honey stomach | no inhibition |  |
| P26 | honey stomach | no inhibition |  |
| P27 | honey stomach | no inhibition |  |
| P35 | honey stomach | no inhibition |  |
| P38 | honey stomach | no inhibition |  |
| P40 | honey stomach | no inhibition |  |
| P59 | honey stomach | no inhibition |  |
| P60 | honey stomach | no inhibition |  |
| P69 | honey stomach | no inhibition |  |
| P70 | honey stomach | no inhibition |  |
| P73 | honey stomach | no inhibition |  |
| P74 | honey stomach | no inhibition |  |
| P80 | honey stomach | < 4 mm |  |
| P81 | honey stomach | no inhibition |  |
| P82 | honey stomach | < 4 mm |  |
| P83 | honey stomach | no inhibition |  |
| P92 | honey stomach | no inhibition |  |
| P94 | honey stomach | no inhibition |  |
| P95 | honey stomach | > 4 mm | P95 |
| P97 | honey stomach | no inhibition |  |
| P100 | honey stomach | > 4 mm | P100 |
| P103 | honey stomach | no inhibition |  |
| P104 | honey stomach | no inhibition |  |
| P106 | honey stomach | no inhibition |  |
| P108 | honey stomach | no inhibition |  |
| P111 | honey stomach | no inhibition |  |
| P10 | mid-gut | < 4 mm |  |
| P25 | mid-gut | > 4 mm | P25 |
| P48 | mid-gut | < 4 mm |  |
| P54 | mid-gut | < 4 mm |  |
| P55 | mid-gut | < 4 mm |  |
