# Supplementary material for: Antimicrobial Activity against Paenibacillus larvae and Functional Properties of Lactiplantibacillus plantarum Strains: Potential Benefits for Honeybee Health
Source: Antibiotics (Basel). 2020 Jul 24;9(8):442. doi: 10.3390/antibiotics9080442 (PMC7460353; doi:10.3390/antibiotics9080442)
Supplement: Supplementary file 1 [file antibiotics-09-00442-s001.zip › supp/Supplementary material/Table S1.pdf]

**L. plantarum strains collection**	**Isolation Source**	**ZOI (mm) agar spot test**	**Selected strains**
P3	beebread	< 4 mm	
P4	beebread	no inhibition	
P5	beebread	< 4 mm	
P7	beebread	< 4 mm	
P8	beebread	> 4 mm	P8
P9	beebread	< 4 mm	
P21	beebread	< 4 mm	
P36	beebread	< 4 mm	
P37	beebread	no inhibition	
P39	beebread	no inhibition	
P57	beebread	< 4 mm	
P61	beebread	no inhibition	
P81	beebread	no inhibition	
P82	beebread	< 4 mm	
P86	beebread	> 4 mm	P86
P87	beebread	< 4 mm	
P88	beebread	< 4 mm	
P92	beebread	< 4 mm	
P94	beebread	< 4 mm	
P95	beebread	< 4 mm	
P97	beebread	< 4 mm	
P101	beebread	no inhibition	
P103	beebread	no inhibition	
P106	beebread	no inhibition	
P108	beebread	no inhibition	
P1	honey stomach	no inhibition	
P3	honey stomach	no inhibition	
P10	honey stomach	< 4 mm	
P15	honey stomach	no inhibition	
P21	honey stomach	no inhibition	
P24	honey stomach	no inhibition	
P26	honey stomach	no inhibition	
P27	honey stomach	no inhibition	
P35	honey stomach	no inhibition	
P38	honey stomach	no inhibition	
P40	honey stomach	no inhibition	
P59	honey stomach	no inhibition	
P60	honey stomach	no inhibition	
P69	honey stomach	no inhibition	
P70	honey stomach	no inhibition	
P73	honey stomach	no inhibition	
P74	honey stomach	no inhibition	
P80	honey stomach	< 4 mm	
P81	honey stomach	no inhibition	
P82	honey stomach	< 4 mm	
P83	honey stomach	no inhibition	
P92	honey stomach	no inhibition	
P94	honey stomach	no inhibition	
P95	honey stomach	> 4 mm	P95
P97	honey stomach	no inhibition	
P100	honey stomach	> 4 mm	P100
P103	honey stomach	no inhibition	
P104	honey stomach	no inhibition	
P106	honey stomach	no inhibition	
P108	honey stomach	no inhibition	
P111	honey stomach	no inhibition	
P10	mid-gut	< 4 mm	
P25	mid-gut	> 4 mm	P25
P48	mid-gut	< 4 mm	
P54	mid-gut	< 4 mm	
P55	mid-gut	< 4 mm	
