# Supplementary material for: Antimicrobial Activity against Paenibacillus larvae and Functional Properties of Lactiplantibacillus plantarum Strains: Potential Benefits for Honeybee Health
Source: Antibiotics (Basel). 2020 Jul 24;9(8):442. doi: 10.3390/antibiotics9080442 (PMC7460353; doi:10.3390/antibiotics9080442)
Supplement: Supplementary file 1 [file antibiotics-09-00442-s001.zip › supp/Supplementary material/Table S2.docx]

|  | **Contact**  **Time (min)** | **Hydrophobicity (%)** | | | | |
| --- | --- | --- | --- | --- | --- | --- |
|  |  | **P8** | **P25** | **P86** | **P95** | **P100** |
| **Xylene** | 15 | 96.5 ± 1.9^Ad^ | 94.6 ± 1.2^Ad^ | 57.1 ± 2.0^Ac^ | 44.9 ± 3.0^Ab^ | 30.0 ± 2.7^Aa^ |
|  | 30 | 97.3 ± 2.1^Ad^ | 98.0 ± 1.1^Ad^ | 58.8 ± 3.1^Ac^ | 46.9 ± 3.0^Ab^ | 33.5 ± 1.4^Aa^ |
|  | 60 | 99.4 ± 0.4^Ad^ | 99.1 ± 0.6^Ad^ | 59.3 ± 1.9^Ac^ | 47.4 ± 1.4^Ab^ | 36.1 ± 2.1^Aa^ |
| **Toluene** | 15 | 95.7 ± 0.9^Ad^ | 95.6 ± 0.6^Ad^ | 31.5 ± 2.0^Ab^ | 47.2 ± 1.3^Ac^ | 19.5 ± 1.1^Aa^ |
|  | 30 | 97.3 ± 1.3^Ad^ | 97.2 ± 1.4^Ad^ | 46.9 ± 1.6^Bb^ | 47.6 ± 2.1^Ac^ | 34.8 ± 1.8^Ba^ |
|  | 60 | 99.6 ± 0.2^Ad^ | 98.7 ± 0.2^Ad^ | 59.1 ± 1.5^Cc^ | 47.8 ± 1.9^Ab^ | 38.2 ± 1.9^Ca^ |
