# Supplementary material for: Antimicrobial Activity against Paenibacillus larvae and Functional Properties of Lactiplantibacillus plantarum Strains: Potential Benefits for Honeybee Health
Source: Antibiotics (Basel). 2020 Jul 24;9(8):442. doi: 10.3390/antibiotics9080442 (PMC7460353; doi:10.3390/antibiotics9080442)
Supplement: Supplementary file 1 [file antibiotics-09-00442-s001.zip › supp/Supplementary material/Table S2.pdf]

| | Contact Time (min) | Hydrophobicity (%) | | | | |
|---|---|---|---|---|---|---|
| | | P8 | P25 | P86 | P95 | P100 |
| **Xylene** | 15 | 96.5 ± 1.9$^{Ad}$ | 94.6 ± 1.2$^{Ad}$ | 57.1 ± 2.0$^{Ac}$ | 44.9 ± 3.0$^{Ab}$ | 30.0 ± 2.7$^{Aa}$ |
| | 30 | 97.3 ± 2.1$^{Ad}$ | 98.0 ± 1.1$^{Ad}$ | 58.8 ± 3.1$^{Ac}$ | 46.9 ± 3.0$^{Ab}$ | 33.5 ± 1.4$^{Aa}$ |
| | 60 | 99.4 ± 0.4$^{Ad}$ | 99.1 ± 0.6$^{Ad}$ | 59.3 ± 1.9$^{Ac}$ | 47.4 ± 1.4$^{Ab}$ | 36.1 ± 2.1$^{Aa}$ |
| **Toluene** | 15 | 95.7 ± 0.9$^{Ad}$ | 95.6 ± 0.6$^{Ad}$ | 31.5 ± 2.0$^{Ab}$ | 47.2 ± 1.3$^{Ac}$ | 19.5 ± 1.1$^{Aa}$ |
| | 30 | 97.3 ± 1.3$^{Ad}$ | 97.2 ± 1.4$^{Ad}$ | 46.9 ± 1.6$^{Bb}$ | 47.6 ± 2.1$^{Ac}$ | 34.8 ± 1.8$^{Ba}$ |
| | 60 | 99.6 ± 0.2$^{Ad}$ | 98.7 ± 0.2$^{Ad}$ | 59.1 ± 1.5$^{Cc}$ | 47.8 ± 1.9$^{Ab}$ | 38.2 ± 1.9$^{Ca}$ |
