# Supplementary material for: Antimicrobial Activity against Paenibacillus larvae and Functional Properties of Lactiplantibacillus plantarum Strains: Potential Benefits for Honeybee Health
Source: Antibiotics (Basel). 2020 Jul 24;9(8):442. doi: 10.3390/antibiotics9080442 (PMC7460353; doi:10.3390/antibiotics9080442)
Supplement: Supplementary file 1 [file antibiotics-09-00442-s001.zip › supp/Supplementary material/Table S3.docx]

| **Time**  **(hours)** | **Auto-Aggregation (%)** | | | | |
| --- | --- | --- | --- | --- | --- |
|  | **P8** | **P25** | **P86** | **P95** | **P100** |
| 1 | 10.9 ± 0.8^Aa^ | 16.0 ± 1.1^Ab^ | 10.7 ± 0.8^Aa^ | 16.0 ± 0.3^Ab^ | 12.3 ± 0.7^Aa^ |
| 2 | 14.2 ± 0.7^Ba^ | 20.0 ± 1.6^Ba^ | 15.2 ± 0.8^Ba^ | 19.5 ± 1.2^Ba^ | 17.8 ± 1.1^Ba^ |
| 5 | 19.4 ± 0.5^Ca^ | 24.8 ± 0.8^Ca^ | 20.3 ± 1.9^Ca^ | 23.0 ± 0.9^Ca^ | 22.3 ± 0.6^Ca^ |
| 24 | 97.8 ± 1.0^Db^ | 99.5 ± 0.2^Cb^ | 78.6 ± 3.6^Da^ | 80.8 ± 0.9^Da^ | 79.2 ± 3.0^Da^ |
