# Supplementary material for: Antimicrobial Activity against Paenibacillus larvae and Functional Properties of Lactiplantibacillus plantarum Strains: Potential Benefits for Honeybee Health
Source: Antibiotics (Basel). 2020 Jul 24;9(8):442. doi: 10.3390/antibiotics9080442 (PMC7460353; doi:10.3390/antibiotics9080442)
Supplement: Supplementary file 1 [file antibiotics-09-00442-s001.zip › supp/Supplementary material/Table S3.pdf]

| Time (hours) | Auto-Aggregation (%) | | | | |
|---|---|---|---|---|---|
| | **P8** | **P25** | **P86** | **P95** | **P100** |
| 1 | $10.9 \pm 0.8^{Aa}$ | $16.0 \pm 1.1^{Ab}$ | $10.7 \pm 0.8^{Aa}$ | $16.0 \pm 0.3^{Ab}$ | $12.3 \pm 0.7^{Aa}$ |
| 2 | $14.2 \pm 0.7^{Ba}$ | $20.0 \pm 1.6^{Ba}$ | $15.2 \pm 0.8^{Ba}$ | $19.5 \pm 1.2^{Ba}$ | $17.8 \pm 1.1^{Ba}$ |
| 5 | $19.4 \pm 0.5^{Ca}$ | $24.8 \pm 0.8^{Ca}$ | $20.3 \pm 1.9^{Ca}$ | $23.0 \pm 0.9^{Ca}$ | $22.3 \pm 0.6^{Ca}$ |
| 24 | $97.8 \pm 1.0^{Db}$ | $99.5 \pm 0.2^{Cb}$ | $78.6 \pm 3.6^{Da}$ | $80.8 \pm 0.9^{Da}$ | $79.2 \pm 3.0^{Da}$ |
